# Synergistic effects of oxypeucedanin and temozolomide on viability, proliferation, apoptosis, and migration of T98G malignant glioblastoma cells

**DOI:** 10.22038/ajp.2025.26224

**Published:** 2025

**Authors:** Amir Abderam, Parisa Yazdi, Farzaneh Abbasinezhad-moud, Matin Shirazinia, Ghazaleh Pouyamanesh, Maryam Shojaee, Fatemeh Ardalan Moghadam Al, Afsane Bahrami

**Affiliations:** 1 *Student Research Committee, Birjand University of Medical Sciences, Birjand, 9717853577, Iran*; 2 *Department of Medical Biochemistry, Faculty of Medicine, Mashhad University of Medical Sciences, Mashhad, Iran*; 3 *Student Research Committee, Mashhad University of Medical Sciences, Mashhad, Iran*; 4 *Department of medical laboratory science, Mashhad branch, Islamic Azad University, Mashhad, Iran*; 5 *Department of Biology, Damghan Branch, Islamic Azad University, Damghan, Iran*; 6 *Clinical Research Development Unit, Imam Reza Hospital, Faculty of Medicine, Mashhad University of Medical Sciences, Mashhad, Iran*; 7 *Clinical Research Development Unit of Akbar Hospital, Faculty of Medicine, Mashhad University of Medical Sciences, Mashhad, Iran*

**Keywords:** Oxypeucedanin, Temozolomide, Glioblastoma, Apoptosis, Migration, Proliferation

## Abstract

**Objective:**

Glioblastoma multiforme (GBM), an aggressive primary brain tumor, distinguished by an invasive growth pattern and resistance to current therapeutic strategy. This study investigates the potential of Oxypeucedanin (OP) as a natural compound to induce apoptosis and inhibit proliferation in T98G GBM cells, either alone or in combination with Temozolomide (TMZ).

**Materials and Methods:**

T98G cells were exposed to OP and TMZ individually and in combination. Then, cell viability (MTT assay), cell proliferation (using trypan blue), mRNA expression (qRT-PCR), Cell cycle and apoptosis (flow cytometry), and migration (wound healing assay) were evaluated.

**Results:**

The viability assays revealed that both OP and less potentially TMZ decreased cell viability in a time- and dose-dependent manner. Notably, the combination of OP and TMZ demonstrated synergistic effects, substantially enhancing apoptosis rates while reducing proliferation, as evidenced by reduced cell growth rates and altered cell cycle distribution towards G2/M arrest. Additionally, gene expression analysis indicated increased *Bax/Bcl-2* ratios and decreased *Ki-67* levels, suggesting enhanced apoptotic susceptibility and lowered proliferation capacity. Furthermore, the wound healing assay confirmed reduced migration in T98G cells, particularly in the combination treatment group.

**Conclusion:**

This study suggests the potential of OP as a complementary therapeutic agent alongside TMZ for GBM treatment.

## Introduction

Glioblastoma multiforme (GBM) is an extremely aggressive primary brain tumor, characterized by its heterogeneous biology, infiltrative nature, and resistance to chemotherapy (Ostrom et al. 2019). Without treatment, patients typically survive for less than three months. Current therapeutic options including chemotherapeutic agents, radiation therapy, and surgical intervention, only modestly extend survival, with long-term survival rates remaining below 10% (Fernandes et al. 2017; Stupp et al. 2009). Temozolomide (TMZ) is the standard first-line chemotherapeutic drug for treatment of GBM; however, its effectiveness is hindered by factors such as the overexpression of O^6^-methylguanine DNA methyltransferase (MGMT) and the existence of various genetic mutations and repair mechanisms in tumor cells (Gajjar et al. 2022; Messaoudi et al. 2015; Singh et al. 2021; Stupp et al. 2005; Stupp et al. 2017). Therefore, recent research has focused on developing new therapeutic agents to overcome this resistance. Previous studies have shown that coumarins possess properties that can counteract TMZ resistance (Pibuel et al. 2021; Sumorek-Wiadro et al. 2020).

Plant-derived phytochemicals which are biologically active compounds, have gained attention as possible therapeutic agents due to their ability to influence various cellular mechanisms associated with GBM development (Baliyan et al. 2025). Furanocoumarin compounds are natural substances that show potential in the treatment of GBM (Huang et al. 2024; Wang et al. 2023). One such compound is oxypeucedanin (OP), derived from plants in the Apiaceae and Rutaceae families. Certain plants containing OP are commonly used in food. Examples include the leaves of *Anethum graveolens*, *Angelica archangelica*, *Prangos* species, and *Ferulago* species, as well as well-known citrus fruits. In traditional medicine, *Angelica dahurica* roots have been utilized in China for relieving toothaches and treating the common cold, while in Iran, the leaves of *Prangos* have been employed as an anthelmintic and carminative remedy (Mottaghipisheh 2021). OP exhibits multiple biological activities, including anti-cancer effects. OP has demonstrated the ability to induce apoptosis in cancer cells with minimal toxicity to normal cells and has possible synergistic potential with conventional chemotherapy drugs like doxorubicin (Escoubas et al. 1992; Mottaghipisheh 2021; Mottaghipisheh et al. 2018; Seo et al. 2013). One of the mechanisms by which OP can reverse multidrug resistance is the inhibition of the efflux pump (Mottaghipisheh et al. 2018). 

As far as we know, no study has examined the therapeutic properties of OP on GBM cells. Furthermore, since coumarins enhance the TMZ-sensitivity of GBM cells and OP exhibits anticancer effects, we designed this study to investigate the potential of OP in inducing apoptosis and inhibiting proliferation and migration in TMZ-resistant T98G GBM cells, emphasizing the need for novel therapeutic strategies in GBM management.

## Materials and Methods

### Chemicals and cell lines

High-glucose Dulbecco’s Modified Eagle’s Medium (DMEM-HG), Non-Essential Amino Acids (NEA), trypsin-EDTA, penicillin-streptomycin, and Fetal Bovine Serum (FBS) were obtained from Gibco (Grand Island, NY, USA). OP was provided by Golexir Pars Company (Mashhad, Iran). The dichloro-dihydro-fluorescein diacetate (DCFDA)/H2DCFDA cellular ROS detection assay kit was sourced from Abcam (Cambridge, UK). Annexin V-FITC assay kits and TMZ were purchased from Cayman Chemical (Michigan, MI, USA). Except when stated otherwise, all other reagents were obtained from Sigma-Aldrich (St. Louis, MO, USA).

The normal Human Foreskin Fibroblast (HFF) cells and T98G GBM cell line were used in current research. To sustain their proliferation, the cells were cultured in T75 filter cap flasks. The T98G cells were maintained in DMEM-HG (containing 2 mM glutamine) plus 1% NEA, while the HFF cells were cultured in DMEM-HG alone. Both media were supplemented with 10% FBS. The cells were kept at 37°C in a humidified incubator with 5% carbon dioxide and minimal light exposure. The culture medium was replaced every 2 days, and passaging was conducted when the cell population reached around 90% confluency.

### Cell culture and viability assay

Cell viability examination was evaluated by the MTT assay. Briefly, T98G (1.2 × 10⁴ cells per well) and HFF (1.5 × 10⁴ cells per well) were plated in 96-well plates and allowed to incubate overnight at 37°C with 5% carbon dioxide. The cells were then exposed to various concentrations of OP (ranging from 0 to 500 μM) and TMZ (ranging from 0 to 1000 μM) for 24, 48, and 72 hr. Following treatment, MTT was introduced into each well and left to incubate for three hours. The formed formazan crystals were then dissolved in DMSO solution, and absorbance was recorded at 570 nm using an Epoch ELISA reader. The values of IC50 were determined utilizing GraphPad Prism® 8.2.1 software.

### Combined drug effect analysis

T98G cells were plated in 96-well plates at a concentration of 1.2 × 10⁴ cells per well and exposed to different concentrations of OP and TMZ. Each treatment was carried out in triplicate, and cell viability was assessed. The combination index (CI) was calculated using CalcuSyn software (Cambridge, UK) to assess whether the drug combinations had synergistic (CI<1), additive (CI=1), or antagonistic (CI>1) effects.

### Cell proliferation assay

Cell proliferation was evaluated by the trypan blue dye exclusion method (Funk and Musa 2021). T98G cells (4 × 10⁴ cells per well) were seeded in 12-well plates and exposed to OP (50 µM), TMZ (260 µM), and a combination of both (50 µM OP + 260 µM TMZ). Following exposure of 24, 48, 72, and 96 hr, cells were trypsinized, mixed with 0.4% trypan blue, and the number of viable and nonviable cells was determined by a hemacytometer. Cell counts were calculated as follows: 

Total cells = Unstained viable cells × dilution factor × 10⁴

In addition, the growth rate of T98G cells were calculated as follow:

Growth Rate (cells/hour) = Ln$\frac{\mathrm{Finall cell number}}{\mathrm{Initial cell number}}$ ÷ Duration

### Annexin V-FITC and cell cycle analysis

T98G cells (2.5 × 10⁵) were seeded in 12-well plates and exposed to OP (50 µM), TMZ (260 µM), and a combination of both (50 µM OP + 260 µM TMZ) for 72 hr. For annexin V-FITC, cells were trypsinized, washed with incubation buffer, stained with annexin V-FITC and propidium iodide (PI), and incubated in the dark for 10 min. 

For the purpose of cell cycle analysis, the cells were first trypsinized, then fixed in 70% ethanol at 4°C for 2 hr. Washed with Phosphate-buffered saline (PBS), they were stained with a solution of PI containing Triton X-100, RNase, PI, and sodium citrate. Flow cytometry was performed on 10,000 cells by a BD FACSCALIBUR™ flow cytometer (Becton Dickinson, USA). For analyzing the data, FlowJo software was utilized.

### Quantitative real-time polymerase chain reaction (qRT-PCR)

The expression of mRNA was assessed using qRT-PCR to evaluate mRNA expression for *Bax*, *Bcl-2*, and *ki67* after RNA extraction and cDNA synthesis from T98G cells treated with OP (50 µM), TMZ (260 µM), and a combination of TMZ and OP (50 µM OP + 260 µM TMZ) for 72 hr as described previously (Qoorchi Moheb Seraj et al. 2022). 

### Scratch wound healing assay

The scratch wound healing assay was conducted to evaluate the anti-migration properties of OP (25 µM), TMZ (130 µM), and their combination (25 µM OP + 130 µM TMZ). The impacts of the drugs on migration of cell were assessed by creating wounds in cell monolayers and observing healing over 2 and 24 hr, with analysis performed using Fiji ImageJ software.

### Measurement of reactive oxygen species (ROS) generation

The impact of OP (50 µM), TMZ (260 µM), and a combination of TMZ and OP (50 µM OP + 260 µM TMZ) on ROS production in T98G cells was evaluated using the H2DCFDA assay following the manufacturer's instructions. The fluorescence intensity was measured by a Victor X5 Multilabel Plate Reader (Excitation/Emission: 485/535 nm).

### Toxicity and pharmacokinetic properties of OP

The toxicity profile of OP was evaluated using the online tool ProTox-II, which assesses various toxicity aspects including cytotoxicity, hepatotoxicity, carcinogenicity and mutagenicity, immunotoxicity, and adverse outcome pathways (Tox21). ProTox-II employs molecular similarity, pharmacophore modeling, fragment analysis, and machine learning algorithms to predict toxicity endpoints. Additionally, computational analyses of OP’s pharmacokinetic properties were conducted using the SwissADME server, focusing on its permeability across the blood-brain barrier, gastrointestinal absorption, designation as a P-glycoprotein (P-gp) substrate, and its inhibitory effects on several cytochrome P450 enzymes (CYP1A2, CYP2C9, CYP2C19, CYP3A4, and CYP2D6).

### Statistical analysis

For data analysis, GraphPad Prism® 8.2.1 was used, applying the Kruskal–Wallis test followed by Dunn’s post hoc test for pairwise comparisons. The threshold for statistical significance was set at p<0.05. The results are presented as the mean to represent the central tendency and the standard deviation to indicate the data distribution.

## Results

### OP and TMZ reduced cell viability in a concentration-dependent manner

**Figure 1:**
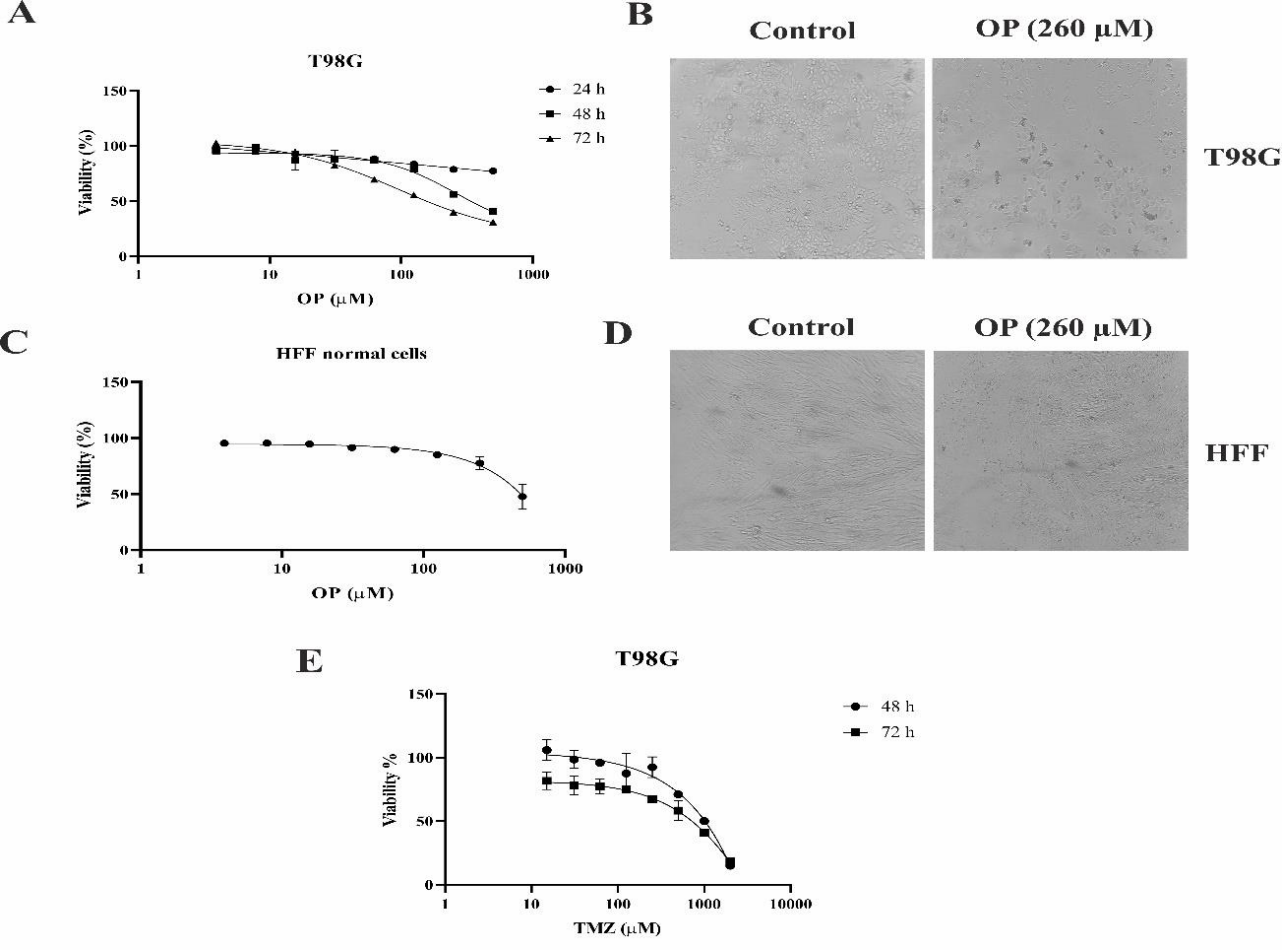
*Cytotoxic effects of (A and C) OP and (E) TMZ at varying doses on T98G and HFF cells. Cell viability following treatment of HFF and T98G cells was evaluated using MTT viability assay method. The phase contrast images of (B) T98G and (D) HFF cells after 48 hr of treatment with IC*_50_ *of OP (260 µM). (10x magnification) (Mean ± SD).*

The viability assay results showed that OP and TMZ could decrease the viability of T98G GBM cells in a time and concentration-dependent manner. As it has been shown in Figure 1E and 1A, both TMZ and OP decreased the viability of T98G cells, with an IC_50_ value of 980 µM and 260 µM following 48 hr of exposure, respectively. Moreover, the IC_50_ value for TMZ and OP following 72 hr exposure was 520 µM and 100 µM, respectively (Table 1).

To evaluate the toxicity of OP on normal cells, we exposed HFF cells to OP for 72 hr. Microscopic images shown in Figure 1D demonstrated that OP at the IC_50_ dose for T98G cells (260 µM) had little impact on HFF cells, with minimal detachment and shrinkage observed. The IC_50_ of OP for HFF cells after 72 hr of treatment was also determined to be about 500 µM (Figure 1C), indicating that OP is less toxic to normal HFF cells compared to cancerous T98G cells.

**Table 1:** *The IC*_50_ *value of TMZ and OP after 24, 48, and 72 hr of T98G and HFF cells treatment*

**Cells**	Compound	24 hr	48 hr	72 hr
**T98G**	TMZ (µM)	-	~ 980	~ 520
**T98G**	OP (µM)	> 500	260	100
**HFF**	OP (µM)	-	-	~ 500

### OP had synergistic effect with TMZ

**Figure 2:**
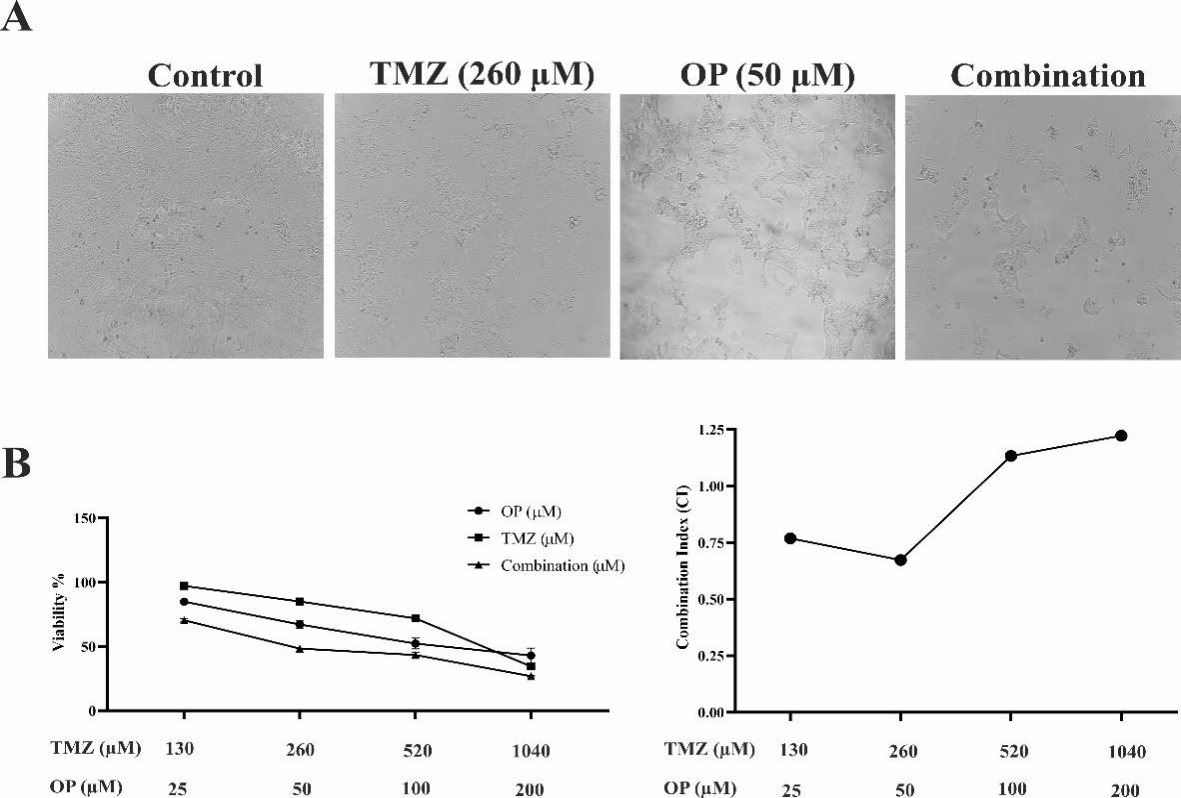
In the T98G cell line, the combined treatment of OP and TMZ resulted in a synergistic effect. (A) Phase-contrast images of T98G cells captured after 72 hr of exposure to OP (50 µM), TMZ (260 µM), and their combination (10x magnification). (B) Cells were cultured with OP and TMZ individually and in combination, demonstrating a significant increase in cytotoxicity with the combined treatment. Data are expressed as mean ± SD. The dose-response relationship was assessed using the CalcuSyn software, and the combination index (CI) was calculated. The CI value quantitatively determines the interaction type, where CI < 1 indicates synergy, CI = 1 represents an additive effect, and CI > 1 suggests antagonism.

The MTT assay‘s results showed that both OP and TMZ exhibited minimal toxicity at doses below the IC_50_. However, when used together at the same concentrations, the combination notably reduced the proliferation of T98G GBM cells. The combination treatment displayed a synergistic effect (CI<1) across all doses tested. Specifically, using 50 µM of OP and 260 µM of TMZ resulted in a low CI value and approximately 50% reduction in cell viability, leading to the selection of these concentrations for further study (Figure 2). 

### OP alone or more potentially in combination with TMZ decreased the proliferation of GBM cells

**Figure 3:**
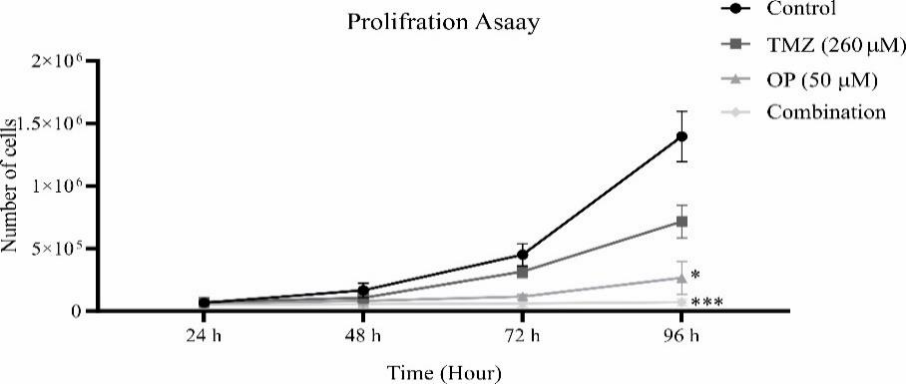
Trypan blue dye exclusion method was used to show T98G cells proliferation following treatment with OP (50 µM), TMZ (260 µM), and a combination of both compared to untreated control group after 24, 48, 72, and 96 hr.

The results of trypan blue dye exclusion method showed that T98G cells proliferate in a time dependent manner with growth rate of 0.042 ± 0.006 cells/hour after 96 hr. However, TMZ and more potently OP decreased the proliferation of T98G cells after 96 hr. A combination OP with TMZ also more potentially and significantly decreased the proliferation of T98G cells compared to control (Figure 3). TMZ and OP could also decrease the growth rate of T98G cells to 0.032 ± 0.006 cells/hour and 0.021 ± 0.014 cells/hour after 72 hr, respectively. The results also showed that a combination of TMZ with OP after 96 hr could more potentially decrease the growth rate of T98G cells to 0.004 ± 0.008 cells/hour (Table 2). 

**Table 2:** The growth rate (Cells/hour) of T98G cells was calculated after 72 and 96 hr following treatment with OP (50 µM), TMZ (260 µM), and a combination of both.

**Groups**	Growth Rate (Cells/hour)	
	24 to 72 hr	24 to 96 hr
**Control**	0.0407 ± 0.008	0.042 ± 0.006
**TMZ (260 µM)**	0.032 ± 0.007	0.032 ± 0.006
**OP (50 µM)**	0.017 ± 0.018	0.021 ± 0.014
**Combination**	0.001 ± 0.016	0.004 ± 0.008

### OP alone or in combination with TMZ increased the amount of apoptotic cells

**Figure 4:**
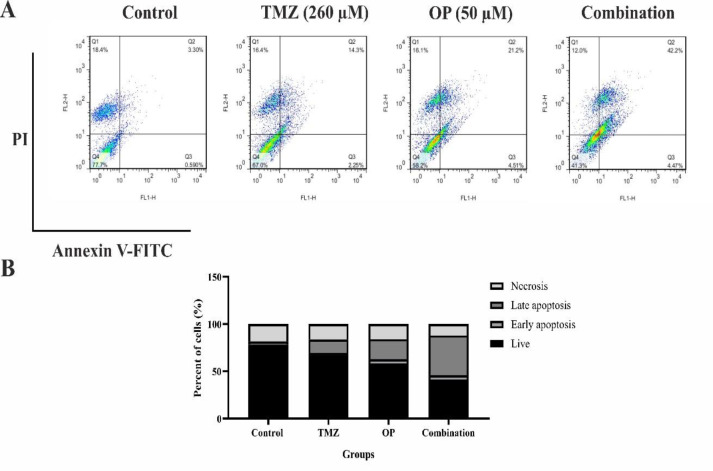
(A) T98G cells were stained with annexin V-FITC and PI, then analyzed via flow cytometry to assess apoptosis and necrosis following treatment with OP (50 µM), TMZ (260 µM), and their combination. The x-axis and y-axis represent annexin V-FITC and PI staining, respectively. The diagram categorizes cells into four quadrants: Q4 (live cells), Q3 (early apoptotic cells), Q2 (late apoptotic cells), and Q1 (necrotic cells). (B) T98G cells exposed to the combined treatment of OP (50 µM) and TMZ (260 µM) exhibited a notable higher proportion of early and late apoptotic cells compared to the control and single-treatment groups.

The annexin V-FITC/PI apoptosis assay was employed to assess the early and late stages of apoptosis in T98G cells after exposure to TMZ, OP, and their combination. Figure 4A illustrates the data, showing that the amount of late apoptotic cells increased from 3.30% in control to 14.3% in TMZ treatment group and 21.2% in OP-treated group. Moreover, when T98G cells were exposed to the combination of TMZ and OP, a considerable elevation in the number of late apoptotic cells (42.2%) was observed (Figure 4B).

### OP alone or in combination with TMZ increased the amount of G2/M cell cycle arrest

**Figure 5:**
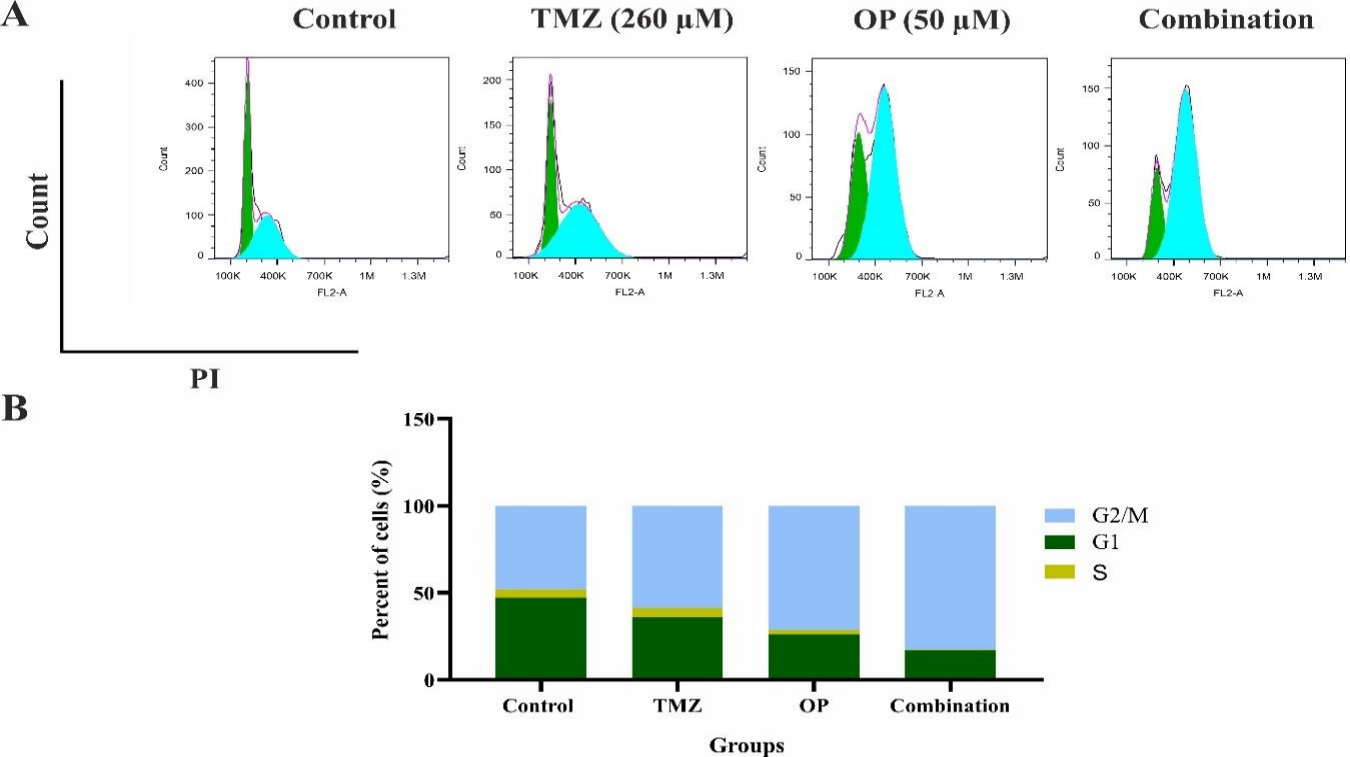
(A) The impact of OP (50 µM), TMZ (260 µM), and their combination on the cell cycle of T98G cells was analyzed after 72 hr of treatment. (B) T98G cells exposed to the combined treatment of OP (50 µM) and TMZ (260 µM) exhibited a considerable increased accumulation in the G2/M phase compared to the control and single-treatment groups, indicating enhanced cell cycle arrest.

The results of the current research showed that treatment of T98G cells with TMZ and OP caused a substantial cell cycle arrest in G2/M Phase (Figure 5B). When T98G cells were treated with TMZ and OP, the amount of cells in G2/M phase of cell cycle from 47.94% in control group reached 58.71% in TMZ and 71.29% in OP-treated group. Moreover, combination therapy of TMZ with OP caused higher accumulation of cells in the G2/M phase. The proportion of cells in combination treatment group reach to 82.53% (Figure 5A). 

### OP alone or in combination with TMZ modulated the mRNA expression of apoptotic and proliferative genes

**Figure 6:**
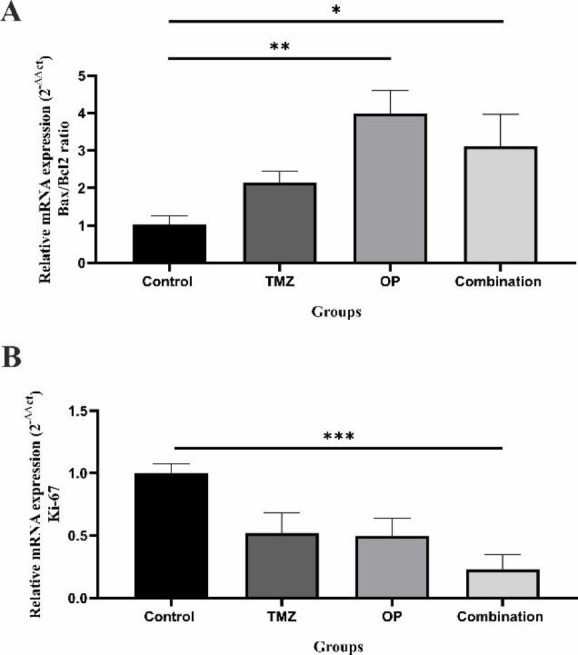
*T98G glioblastoma cells were exposed to OP (50 µM), TMZ (260 µM), or their combination for 72 hours. Following treatment, cells were collected for qRT-PCR analysis to evaluate the gene expression levels of (A) the *Bax/Bcl-2* ratio and (B) *Ki-67. Data are expressed as mean ± standard deviation. (*p<0.05, **p<0.01, and ***p<0.001 compared to the control). (n=4).

RT-PCR analysis was conducted to examine the key gene expression related to cell proliferation and cell apoptosis. The *Bax/Bcl2* mRNA expression ratio was used as an indicator of apoptosis susceptibility, and its level significantly increased following treatment with OP alone and in combination of OP with TMZ (Figure 6A). In addition, combination of TMZ with OP significantly decreased the *Ki-67* mRNA expression, a well-known marker of cell proliferation (Figure 6B). 

### Combination of OP with TMZ decreased the migration and proliferation of T98G cells

**Figure 7:**
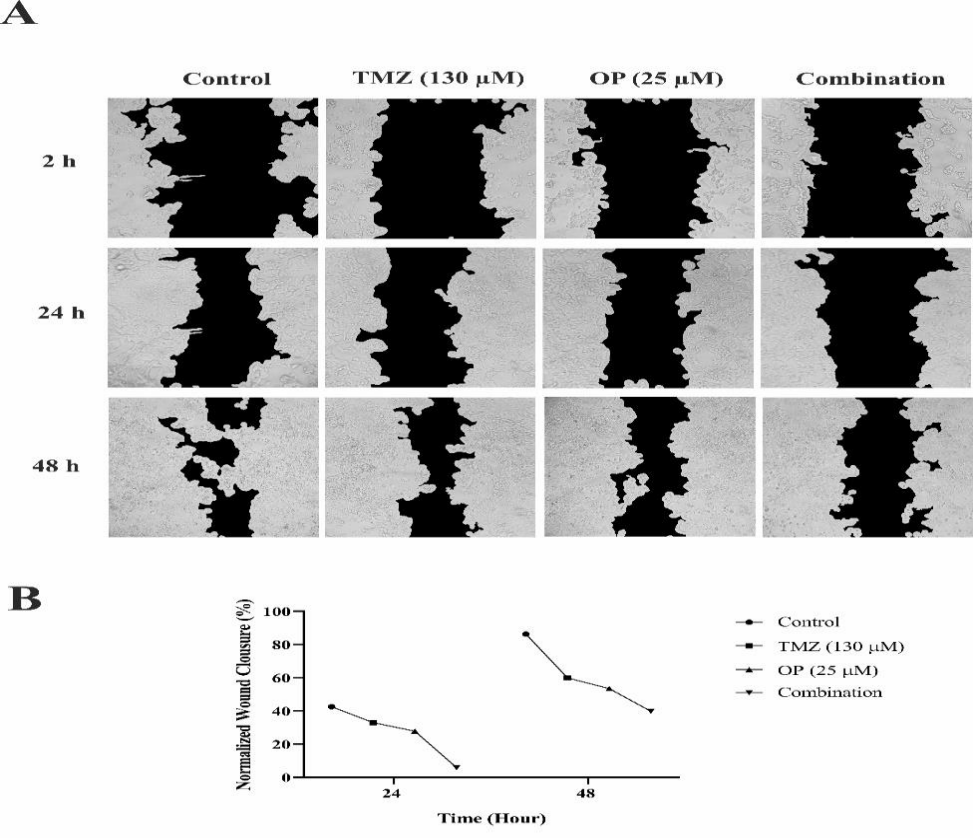
The impact of OP (25 µM), TMZ (130 µM), and their combination on T98G cell migration was evaluated using a wound-healing assay. (A) Phase-contrast microscopy was used to capture images of wound closure. (B) After 24 and 48 hr, the results indicated that combination therapy substantially reduced cell migration. The data were normalized to account for cell proliferation percentages.

The scratch wound healing assay was utilized to evaluate the effects of OP, TMZ, and the co-treatment of them on T98G cell migration. As depicted in Figure 7A, all groups, including OP, TMZ, and their combination, led to a reduction in T98G cell migration after 24 hr (Figure 7B). Moreover, co-treatment more notably decreased the migration of T98G cells after 24 and 48 hr. 

### The co-treatment of OP and TMZ induced a significant increase in ROS production

**Figure 8:**
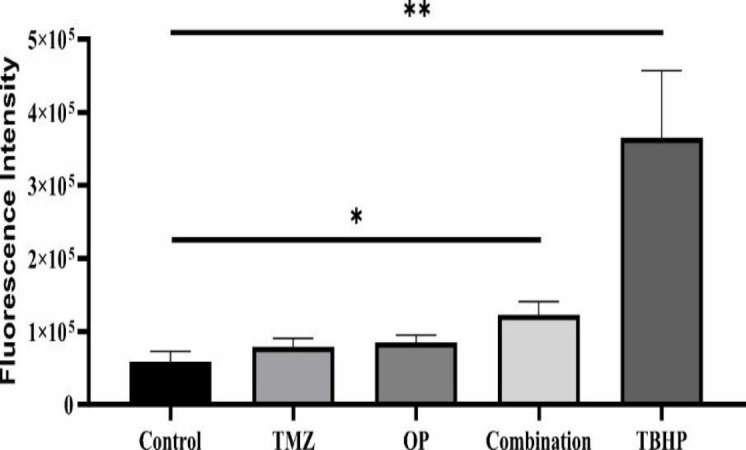
*After 4 hr of treatment, the impact of OP (50 µM), TMZ (260 µM), and their combination on reactive oxygen species (ROS) generation in T98G cells was assessed using DCFDA, a fluorogenic dye. Additionally, 100* *µ* M of tert-Butyl hydroperoxide (TBHP) was used as a positive control. The findings demonstrated that ROS production was significantly elevated in the combination treatment group compared to the control (*p<0.05, **p<0.01). (n=3).

To evaluate the role of ROS in cytotoxicity induced by TMZ, OP, or their co-treatment, we evaluated ROS levels after 4 hr. The results presented in Figure 8 indicated that neither OP nor TMZ significantly affected ROS production. However, treatment of T98G cells with the combination of both compounds for 4 hr led to a significant increase in ROS levels compared to the control. Likewise, Tert-Butyl hydroperoxide (TBHP), used as a positive control, also caused a significant elevation in ROS generation.

### Toxicity and pharmacokinetic properties of OP

**Figure 9:**
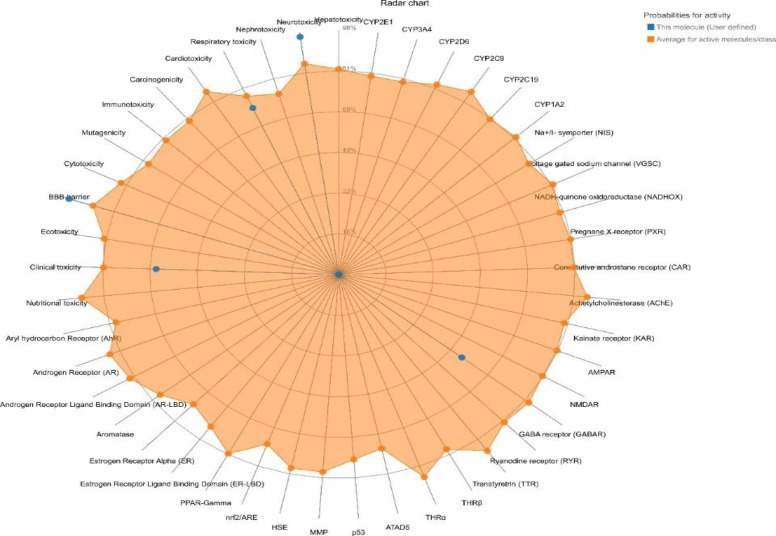
The toxicity radar chart illustrate the confidence of positive toxicity results for OP in blue compared to the average for active molecule of its class in orange.

The online server ProTox-II was utilized to predict several toxicity metrics of OP. OP falls into the Class 5 toxicity category, with a median oral lethal dose (LD50) of 3800 mg/kg. Additionally, as shown in the radar chart presented in Figure 8, OP showed active toxicity with probability more than 0.7 for Blood brain barrier (BBB)-barrier-toxicity and neurotoxicity. With lower probability than 0.7 it showed to have probable respiratory and clinical toxicity (Figure 9).

The pharmacokinetic profile of OP was also analyzed, revealing that it exhibits high gastrointestinal absorption and is anticipated to cross the BBB. OP was also predicted to be the inhibitor of P-gp substrate and cytochrome P450 enzymes (Table 3). Also, the amount of topological polar surface area (TPSA) and Consensus Log Po/w was predicted to be 65.11 and 2.72, respectively.

**Table 3:** The pharmacokinetic properties of oxypeucedanin.

GI absorption	BBB permeant	P-gp substrate	CYP1A2 inhibitor	CYP2C19 inhibitor	CYP2C9 inhibitor	CYP2D6 inhibitor	CYP3A4 inhibitor
**High**	Yes	Yes	Yes	Yes	Yes	Yes	Yes

GI, Gastrointestinal; P-gp, P-glycoprotein; CYP, cytochrome P450

## Discussion

GBM is a highly malignant brain tumor that causes significant challenges in oncology due to its resistance to conventional therapies. The standard treatment, TMZ, often faces issues of efficacy due to the tumor’s aggressive biology and the resistance development (Binabaj et al. 2018; Vahedi et al. 2023). Previous studies have suggested that OP, a coumarin derivative, may enhance treatment outcomes for GBM (Almohammed et al. 2024; Choi et al. 2011; Kim et al. 2007; Liu et al. 2016). The current research sheds light on the potential beneficial effects of OP and TMZ-OP co-treatment on the GBM cell line.

In initial assessments, OP demonstrated cytotoxic effects on GBM cells, with an IC50 value of approximately 260 µM over 48 hours. In contrast, its IC50 for normal cells was around 500 µM, indicating a selective effect against tumor cells compared to normal fibroblast cells. This specificity aligns with previous studies demonstrating OP’s ability to inhibit a variety of malignancies, including colon and neuroblastoma cancer cells (Al-shuwaili et al. 2023; Almohammed et al. 2024; Choi et al. 2011). In addition, combination therapy with OP and TMZ significantly reduced GBM cell viability, suggesting its potential as an adjunct treatment for GBM.

The mechanism of OP’s anti-cancer effect appears to involve modulation of the apoptotic pathways, specifically through the Bcl-2 family of proteins. The research indicated that treatment with OP increased the *Bax/Bcl-2* mRNA expression ratio, suggesting a promotion of apoptosis (Al-shuwaili et al. 2023; Qian et al. 2022). Additionally, the combination of OP and TMZ was shown to enhance reactive oxygen species (ROS) production, linking oxidative stress to apoptotic signaling. This was also evident through enhanced apoptotic activity, as indicated by an increased population of apoptotic cells in the flow cytometry analysis. In line with our findings, a study that used a nano-chitosome form of OP on the colorectal cancer cell line HT-29 found that treatment with nano-chitosome-OP induced apoptosis (verified by AO/PI staining) by increasing the expression of caspase-3 and caspase-9 (Almohammed et al. 2024).

Moreover, accumulation of cells in the G2/M phase was induced by OP treatment, which disrupts the rapid proliferation characteristic of GBM cells. This regulation is crucial, given that tumor cells often exhibit dysregulated cell division, leading to genomic instability. Previous studies corroborate these findings by displaying similar effects of OP in other cancer types (Kang et al. 2009; Park et al. 2020). Furthermore, based on previous evidence, OP-induced G2/M phase cell cycle arrest was linked to the reduced expression of checkpoint proteins such as cyclin E, cyclin B1, cdc25c, and cdc2 along with the increased expression of p-chk1 (Park et al. 2020). 

The *Ki-67* expression, a marker for cellular proliferation, was significantly reduced when GBM cells were treated with the OP and OP-TMZ co-treatment. This reduction further emphasizes the synergistic potential of OP in combination with TMZ for effective GBM management (Armocida et al. 2020; Mrouj et al. 2021). A synergistic effect of OP on growth inhibition was observed in hepatoma cells, where co-treatment with OP and gemcitabine suppressed the proliferation of SK-Hep-1 cells (Park et al. 2020).

Notably, OP’s potential ability to inhibit P-glycoprotein (P-gp) and cytochrome P450 enzymes could also contribute to overcoming drug resistance in GBM treatment. OP counteracts P-gp-mediated drug transport by reducing the mRNA levels of P-gp and its subsequent protein expression, as well as inhibiting P-gp activity (Dong et al. 2018). Alongside impacting cell proliferation and apoptosis, OP markedly diminished the migratory capabilities of T98G cells, which is crucial considering the invasive nature of GBM (Liu et al. 2016).

While promising, the study acknowledges its limitations, as all experiments were carried out in vitro (T98G cell lines), which may not completely simulate the tumor microenvironment in vivo. Potential organ toxicity, particularly neurotoxicity, necessitates further investigation in a living model. Additionally, exploring the long-term impacts and resistance mechanisms of OP and TMZ in GBM therapy remains essential for advancing treatment strategies.

This study suggests OP as a potential adjunct therapy for GBM, demonstrating significant anti-cancer effects, particularly when combined with TMZ. The findings invite further exploration into its utility in clinical settings, aiming at optimizing outcomes for GBM patients.

## Research Involving Humans and Animal Statement

The research does not involve humans and animals.
